# Acetate and hypertonic stress stimulate vacuole membrane fission using distinct mechanisms

**DOI:** 10.1371/journal.pone.0271199

**Published:** 2022-07-14

**Authors:** Zeynep Derin Gokbayrak, Dipti Patel, Christopher Leonard Brett

**Affiliations:** Department of Biology, Concordia University, Montreal, Quebec, Canada; Kindai University: Kinki Daigaku, JAPAN

## Abstract

Vacuoles in plants and fungi play critical roles in cell metabolism and osmoregulation. To support these functions, vacuoles change their morphology, e.g. they fragment when these organisms are challenged with draught, high salinity or metabolic stress (e.g. acetate accumulation). In turn, morphology reflects an equilibrium between membrane fusion and fission that determines size, shape and copy number. By studying *Saccharomyces cerevisiae* and its vacuole as models, conserved molecular mechanisms responsible for fusion have been revealed. However, a detailed understanding of vacuole fission and how these opposing processes respond to metabolism or osmoregulation remain elusive. Herein we describe a new fluorometric assay to measure yeast vacuole fission in vitro. For proof–of–concept, we use this assay to confirm that acetate, a metabolic stressor, triggers vacuole fission and show it blocks homotypic vacuole fusion in vitro. Similarly, hypertonic stress induced by sorbitol or glucose caused robust vacuole fission in vitro whilst inhibiting fusion. Using wortmannin to inhibit phosphatidylinositol (PI) -kinases or rGyp1-46 to inactivate Rab–GTPases, we show that acetate stress likely targets PI signaling, whereas osmotic stress affects Rab signaling on vacuole membranes to stimulate fission. This study sets the stage for further investigation into the mechanisms that change vacuole morphology to support cell metabolism and osmoregulation.

## Introduction

Morphology of most organelles is determined by membrane fusion and fission (also called fragmentation). These include mitochondria, chloroplasts, the Golgi apparatus, peroxisomes and organelles of the endocytic pathway including endosomes and metazoan lysosomes, or vacuoles in yeast and plants [1–5]. These opposing processes drive changes in organelle size, number and shape for cellular responses to environmental changes or signaling events or for organelle inheritance during cell division. Endosomes, lysosomes and vacuoles rely on cycles of heterotypic fusion and fission for anterograde and retrograde membrane trafficking through the endocytic pathway [4, 6]. Large numbers of endosomes, lysosomes or vacuoles generated through fission produce enough to be deposited throughout the cell as required for diverse functions, including cell signaling, plasma membrane repair and intra-organelle communication [7, 8]. Enlargement of lysosomes and vacuoles rely on fusion to accommodate autophagy, synchronized with changes in amino acid metabolism by TOR (Target Of Rapamycin) signaling–a key regulator of cell metabolism [9]. Whereas other metabolic perturbations correlate with vacuole fission such as accumulation of acetate, a key intermediary metabolite and conjugate base of acetic acid [10, 11]. In plants and yeast, vacuoles also act as water reservoirs for cytoplasmic osmotic homeostasis required for survival when challenged with high salinity or draught [12]: Vacuoles fill with water, swell and fuse when environmental water is abundant (hypotonic) and release water, shrink and fragment–undergo fission–when water is scarce (hypertonic conditions).

Most knowledge of the molecular machinery underlying organelle membrane fission and fusion was gleaned by studying the budding yeast lysosomal vacuole as a model. *Saccharomyces cerevisiae* cells typically contain 2–5 vacuoles that undergo regulated cycles of membrane fission and fusion [2, 13]. Because they are relatively large (0.5–3 μm diameter) and can be exclusively stained with many vital dyes (e.g. FM4-64), vacuole morphology is easily assessed by fluorescence microscopy [14]. Vacuoles are easily purified permitting further biochemical study of organelle membrane fusion and fission in vitro [15]. Yeast is a genetically tractable model system, permitting genetic analysis as well [16]. Using this system, it was discovered that the mechanisms underpinning these opposing processes are highly coordinated, as one must dominate to effectively change and retain morphology, e.g. to increase copy number, fission is stimulated whilst fusion is blocked, e.g. [17–19]. The basis of homotypic vacuole fusion has been resolved with incredible molecular precision [20]. However, vacuole or organelle fission is less understood.

**Fig 1:**
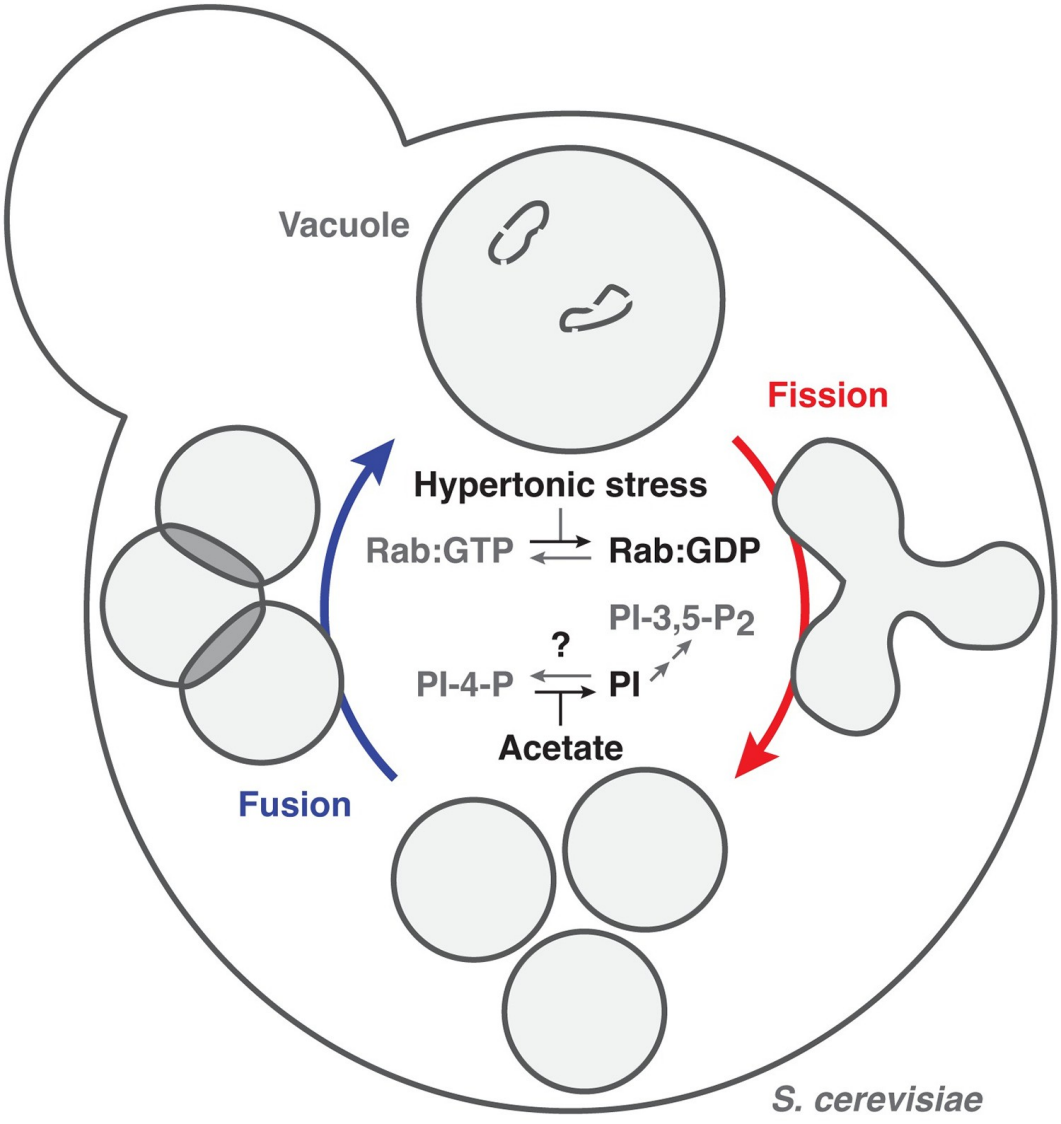
Working model of vacuole morphology affected by metabolic or osmotic stress. Illustration depicting molecular potential interactions between Rab-GTPase and phosphatidylinositol signaling underlying vacuole fission and fusion triggered by acetate or hypertonic stress. Acetate likely inhibits a PI4-kinase whereas hypertonic stress targets Rab-GTPase inactivation to promote vacuole fission over fusion.

Within live yeast cells, vacuoles fragment during the cell cycle and in response to hypertonic stress, oxidative stress, or TOR signaling stimulated by ER stress [2, 17, 21, 22]. Through in vivo and in vitro analysis, it was shown that vacuole fission is a two-step asymmetrical process that requires phosphoinositol-3,5-diphosphate (PI-3,5-P_2_) generated from phosphoinositol-3-phosphate (PI-3-P) on the cytoplasmic face of the vacuole lipid bilayer by Fab1, a PI-3-P5 kinase (or PIKfyve in mammals), in complex with Vac14, Vac7 and Fig 1 [10, 21, 23, 24]. Also implicated in this process is the H^+^-electrochemical gradient maintained by the V-type H^+^-ATPase [10, 21, 22, 25], which likely occurs downstream of Fab1 as its activity is supported by PI-3,5-P_2_ [26, 27]. The process is thought to culminate with lipid bilayer scission by the dynamin-like GTPase Vps1 in coordination with the PROPPIN Atg18, which binds to the Fab1-complex and responds to PI-3,5-P_2_ [25, 28–30].

A decrease in lumenal volume is also necessary to accommodate perimeter membrane collapse and constriction at sites of scission. Currently, it is not entirely clear how this occurs, but insight has been gleaned by experimentally applying hypertonic stress, to drive water out of the vacuole lumen. From these studies, it was revealed that Vac14 is required for activation of Fab1 in response to a decrease in organelle volume induced by hypertonic stress [21]. Recently, Ivy1, an inhibitor of Fab1, inverted BAR (I-BAR) protein and effector of the Rab-GTPase Ypt7 was also implicated in this response [31]: When Ypt7 is inactivated by hypertonic stress [32], fusion is halted and it disengages Ivy1. By also possibly sensing a change in membrane lipid packing or lateral tension induced by loss of lumenal volume, Ivy1 then releases Fab1, activating it to generate PI-3,5-P_2_ and drive fission. Thus, the coordination of PI signaling and Rab-GTPase activity seems important for balancing vacuole fission and fusion. However, how these molecular mechanisms respond to osmotic or metabolic stressors to drive changes in vacuole morphology important for cell physiology remains largely elusive.

Herein we developed a new in vitro vacuole membrane fission assay and test effects of acetate or osmotic stress. Wortmannin to target PI signaling or rGyp1-46 to target Rab-GTPase activity were applied to begin understanding the basis of vacuole morphology regulation.

## Results and discussion

### A new assay to measure vacuole membrane fission in vitro

**Fig 2:**
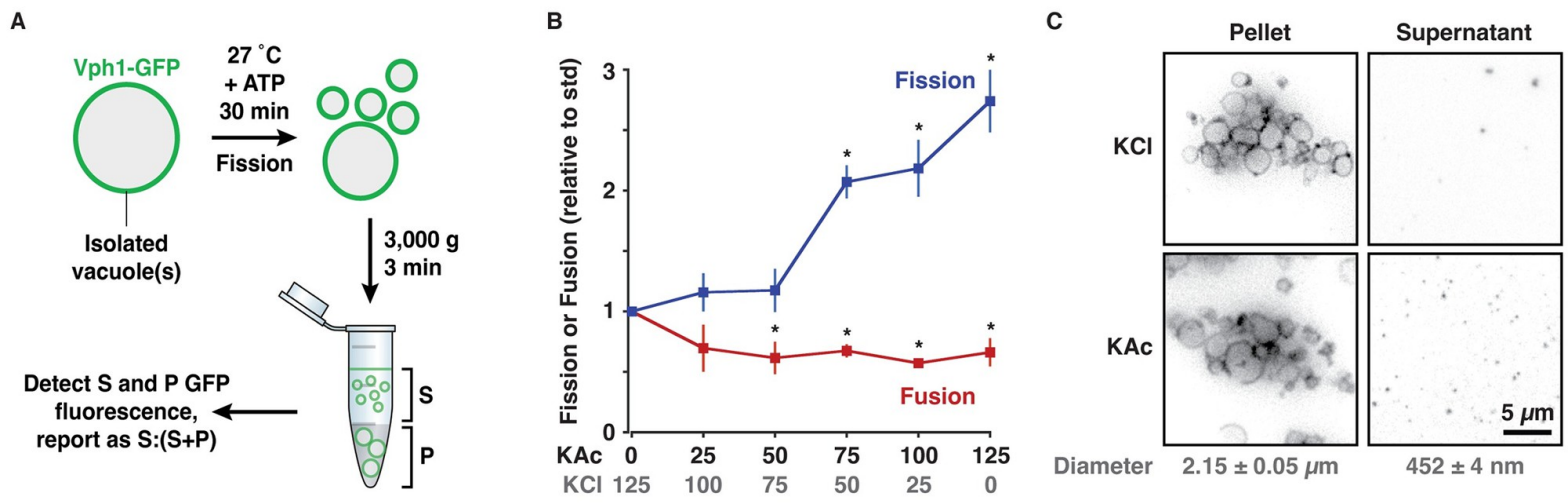
A new, simple cell-free vacuole membrane fission assay. (A) Cartoon depicting new fluorometric in vitro vacuole membrane fission assay. S, supernatant; P, Pellet. (B) Homotypic fusion or fission of isolated vacuoles in the presence of increasing concentrations of KAc in place of KCl. Means ± S.E.M. shown and P < 0.05 (*) as compared to standard conditions (125 mM KCl). (C) Micrographs of fission reactions containing vacuoles isolated from Vph1-GFP expressing yeast cells conducted in the presence of 125 mM KAc or KCl. Supernatants (containing fission products) and pellets (containing large vacuoles) are shown. Means ± S.E.M of vacuole diameters measured by quasi-elastic light scattering are indicated for each fraction.

Researchers currently rely on fluorescence microscopy-based, semi-quantitative assays to estimate numbers of vacuole fission products formed. For example, the number of BODIPY FL-DHPE–stained small (< 0.6 μm diameter), medium (0.6–1.5 μm) and large (≥ 1.5 μm) vacuoles are manually counted using micrographs of in vitro vacuole fission reactions and the fraction of small vacuoles is reported as a fragmentation index [10]. As an alternative, we designed a new assay that involves separating smaller products of fission from larger vacuole precursors using low-velocity differential centrifugation (Fig 2A). To eliminate the need to stain vacuole membranes for detection (with FM4-64 or BODIPY FL-DHPE for example), we isolated vacuoles from yeast cells expressing GFP-tagged Vph1, the stalk domain of the V-type H^+^-ATPase. Vph1-GFP is uniformly distributed on vacuole membranes [33, 34], and thus should decorate fission precursor and product membranes at equal density. Using a plate-reading fluorometer, we measured GFP fluorescence in the supernatant and report the ratio of background-subtracted supernatant fluorescence over total fluorescence (recorded from reactions spared from centrifugation) as a measure of vacuole fission in vitro.

We first conducted this new assay under conditions previously shown to drive vacuole fission in vitro: Freshly isolated vacuoles were incubated at 27 °C for 30 minutes, ATP added as an energy source, and KAc added in place of KCl [10]. As expected, gradually replacing KCl with KAc stimulates vacuole fission in vitro (Fig 2B), whereby complete replacement induced a 2.7–fold increase in fission, consistent with previous findings obtained by using a microscopy-based assay (see Fig 2B in [10]). However, purified cytosol was not required for robust vacuole fission in our hands, suggesting the underlying machinery co-purifies with the organelles. Membrane fusion and fission are opposing mechanisms [17]. Thus, for organelle fragmentation to persist as observed, we hypothesized that vacuole fusion must be inhibited by KAc. To test this hypothesis, we used a lumenal content mixing assay and found that homotypic vacuole fusion was inhibited by KAc replacement in vitro (Fig 2B), confirming that when fission is stimulated, fusion is blocked.

To verify that we separated smaller from larger vacuoles, we imaged pellets and supernatants by HILO microscopy in the presence of 125 mM KCl, when fission is inhibited, or 125 mM KAc when fission is stimulated (Fig 2C). As expected, large vacuoles were observed in the pellet and smaller vacuoles in the supernatant. This was confirmed by measuring vacuole size by quasi-elastic light scattering, whereby vacuole diameter was 4.6 times smaller in the supernatant than in the pellet (Fig 2C). The diameter of the fission products (0.452 ± 0.004 μm) was consistent with a previous estimate (0.45 ± 0.27 μm) obtained from electron micrographs of vacuole fission reactions [10]. When we counted puncta using micrographs of these samples, we found 2.84 ± 0.16 (n ≥ 7) times more GFP-positive vesicles in the supernatant of reactions conducted in the presence of KAc as compared to KCl, consistent with data acquired by fluorometry (Fig 2B). In all, these results validate use of this assay to measure vacuole membrane fission in vitro. We also replicate findings demonstrating that replacing chloride ion with acetate is sufficient to tip the balance towards vacuole fission in vitro.

### Hypertonic shock stimulates vacuole fission in vitro

**Fig 3:**
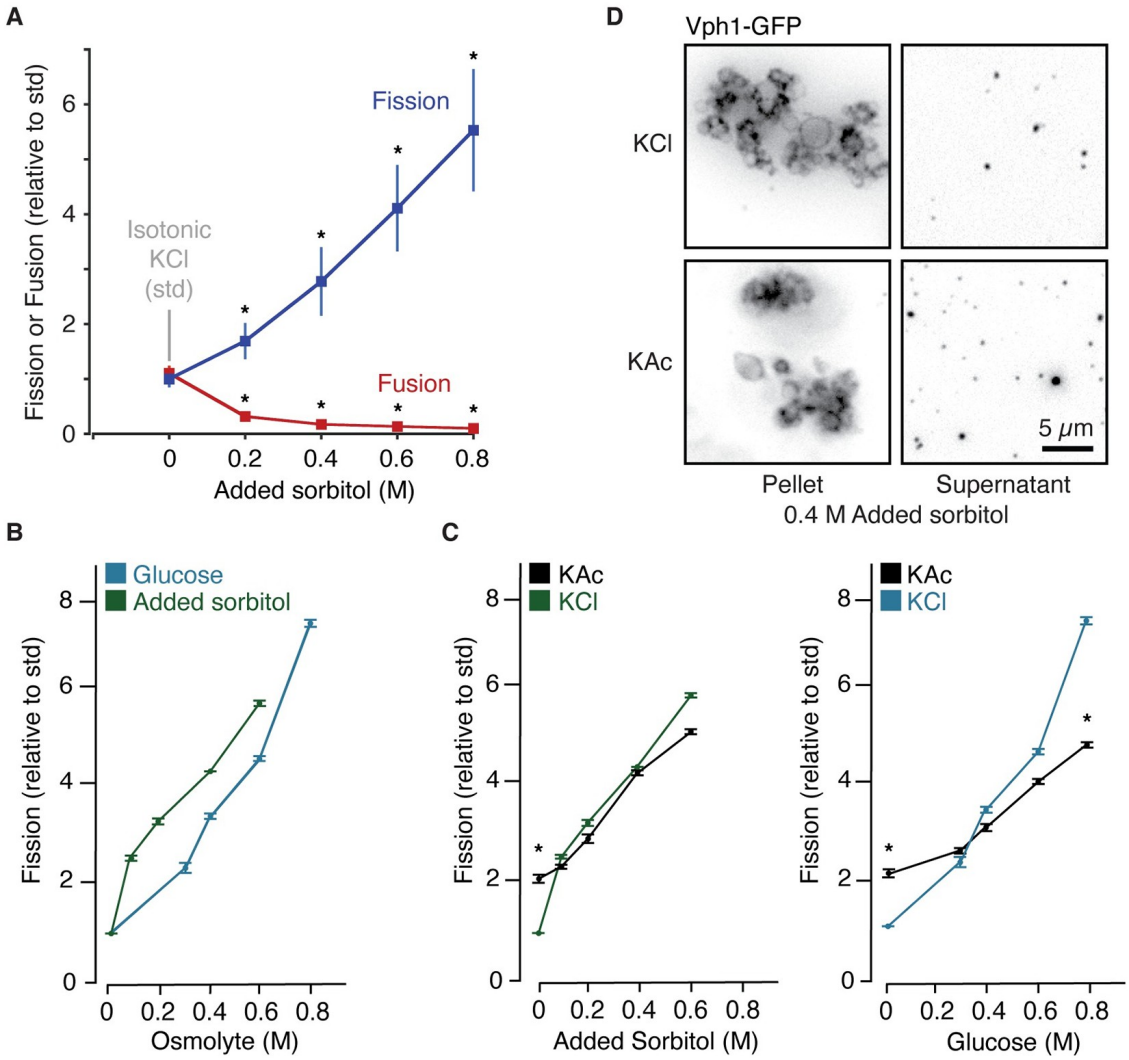
Effects of KAc and hypertonic stress on vacuole fission. (A) Homotypic fusion or fission of isolated vacuoles in the presence of KCl and increasing concentrations of sorbitol added to standard, isotonic conditions (which already contains 0.2 M sorbitol). (B, C) Fission of isolated vacuoles in the presence of either glucose or added sorbitol and 125 mM KCl (B) or KAc (C). Means ± S.E.M. shown and P < 0.05 (*) as compared to standard, isotonic conditions (125 mM KCl, 200 mM sorbitol). (D) Micrographs of fission reactions containing vacuoles isolated from Vph1-GFP expressing yeast cells conducted under hypertonic conditions (0.4 M added sorbitol) in the presence of 125 mM KAc or KCl. Supernatants (containing fission products) and pellets (containing large vacuoles) are shown.

Like plants, *S*. *cerevisiae* use their vacuoles as cellular water reservoirs for cytoplasmic osmotic homeostasis, ensuring survival when exposed to high salinity [12]. This hypertonic environmental stress draws water from lumen of vacuoles and stimulates membrane fission in live yeast cells [17]. However, this was not replicated in vitro when mild hypertonic stress was applied by increasing sorbitol, a sugar alcohol and nonionic osmolyte, from 200 mM to 300 mM in the fission reaction buffer [10]. On the other hand, higher concentrations of sorbitol were reported to block homotypic vacuole fusion in vitro, mimicking cell physiology [32]. Thus, we tested effects of increasing sorbitol concentrations up to 1 M on vacuole fission in the presence of KCl using this new in vitro assay. We found that fission increases proportionally with sorbitol concentration (Fig 3A), whereby 1 M sorbitol shows a 5.6-fold increase in fission as compared to isotonic conditions (200 mM sorbitol, 125 mM KCl). We also confirmed that fusion is inhibited by increasing sorbitol concentrations (Fig 3A), again demonstrating that these processes are inversely regulated.

To demonstrate that this effect was caused by hypertonic stress, as opposed to other chemical properties of sorbitol, we repeated the experiment with glucose, a sugar, key metabolite and organic osmolyte shown to accumulate in the cytoplasm of plant and yeast cells exposed to high salinity [35]. Like sorbitol, increasing amounts of glucose stimulated vacuole fission in vitro in the presence of KCl (Fig 3B). Equimolar concentrations of glucose or sorbitol elicited similar responses (P ≥ 0.13 at each concentration studied), suggesting chemical composition is unimportant and changes in osmolarity are responsible. In support, we observed similar effects when KCl was replaced with KAc (Fig 3C) but noticed that acetate significantly diminished effects of fission only under extreme hypertonic stress (0.8 M glucose). We imaged some of these fission reactions by HILO microscopy (Fig 3D) to confirm that these conditions were not causing vacuole lysis or permitting larger vacuoles to contaminate the supernatant. Of note, this response is organelle–autonomous suggesting that changes in vacuole morphology observed in vivo may not require canonical signaling mechanisms found in the plasma membrane and cytoplasm that sense and respond to environmental osmotic stressors, e.g. the Hog1 pathway [36]. Although speculative, the most likely explanation for this observation is that hypertonic stress drives water efflux from the vacuole lumen, which in turn reduces organelle volume perhaps needed to reshape the membrane prior to fission.

### Acetate and hypertonic stress target different fission mechanisms

Both acetate and hypertonic stress trigger net fragmentation of vacuoles. However, when stressors were applied simultaneously, we noticed that the effect of glucose on fission was suppressed by acetate at high concentrations when compared to chloride (0.8 M; Fig 3C). This observation suggests that (1) chloride ion is needed to optimize vacuole fission is response to hypertonic stress, consistent with its established role in cell volume regulation; and (2) these two stressors may target different fission mechanisms. To further investigate the latter, we targeted PI signaling and Rab-GTPase activity as both are implicated to function in the early stages of fusion and fission [37–39].

**Fig 4:**
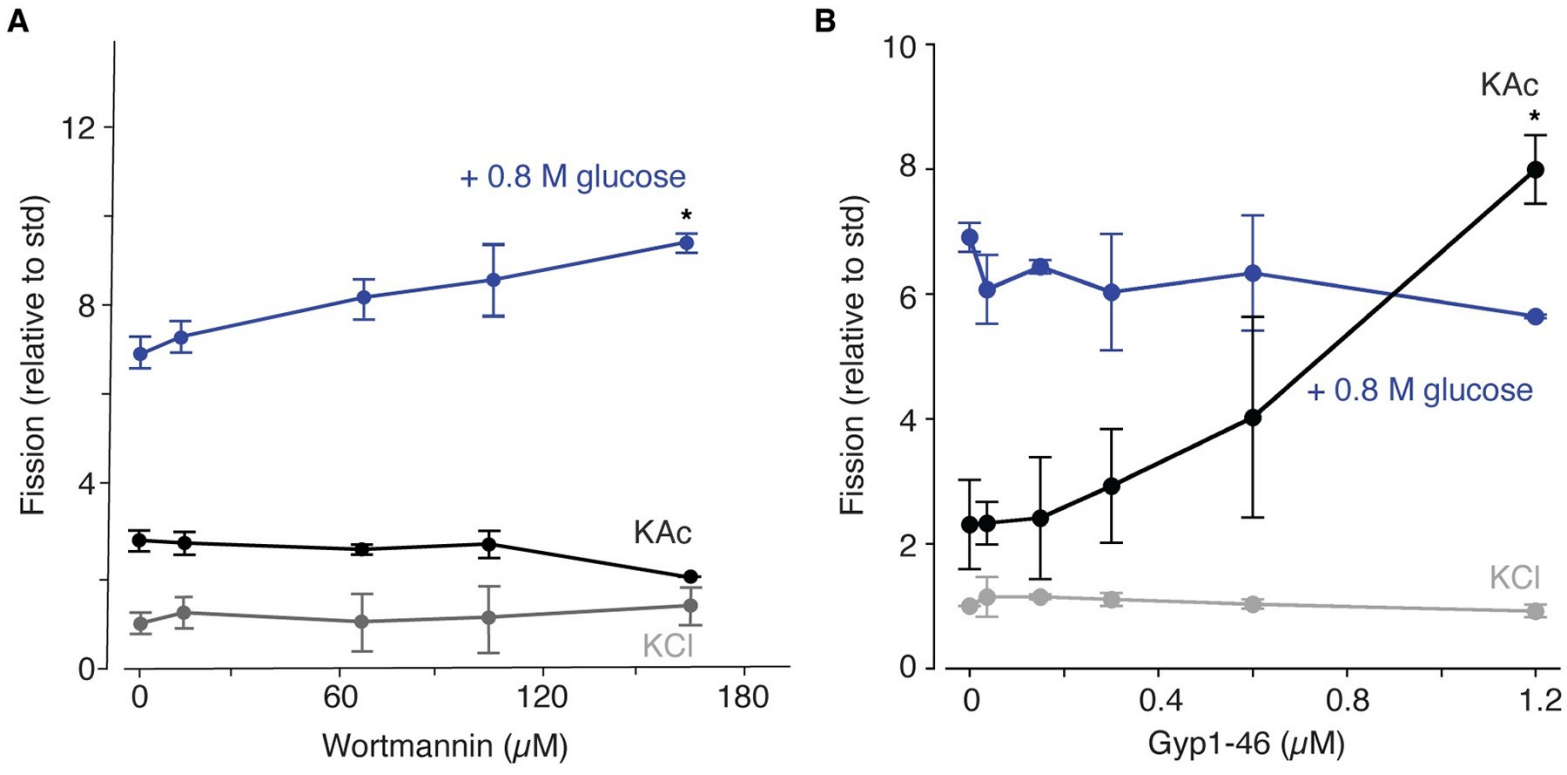
Effects of wortmannin or rGyp1-46 on vacuole fission triggered by acetate or hypertonic stress. Fission of isolated vacuoles in the presence of increasing concentrations of (A) wortmannin or (B) the Rab-GTPase inhibitor rGyp1-46. Fission reactions contained either 125 mM KAc in place of KCl or 125 mM KCl with 0.8 M glucose. Means ± S.E.M. shown.

We first examined PI signaling by acutely inhibiting PI-4-P synthesis in vitro using the PI-kinase inhibitor wortmannin. Although it blocks mammalian PI3-kinase activity, the yeast type III PI3-kinase Vps34 (the only PI3-kinase in *S*. *cerevisiae*) is insensitive to this drug [40]. Rather, it has been reported to block PI-4-P synthesis by the type II PI4-kinase Stt4 [41], and is thought to target orthologous PI4-kinases including Lsb6 found on vacuole membranes [42]. This inhibitor was used instead of a genetic approach, i.e. knocking out STT4, because of anticipated pleiotropic effects given that PI-4-P and PI-4,5-P_2_ are important lipids for signaling at the plasma membrane and influence other vacuole functions [37, 43–45]. We find that increasing concentrations of wortmannin have no effect on fission stimulated by KAc (Fig 4A), suggesting that perhaps a PI4-kinase is already inhibited under these conditions rendering wortmannin ineffective. Importantly, we show that the concentrations of wortmannin used are bioactive as it further stimulated fission in the presence of glucose and KCl (Fig 4A). However, adding wortmannin to reactions without applying a stressor did not significantly affect vacuole fission (under isotonic, KCl conditions; Fig 4A). These results suggest that hypertonic stress does not likely target PI-4-P to induce fission, consistent with previous reports, e.g. [21]. However hypertonic stress facilitates fission by wortmannin perhaps by priming fission mechanisms and/or disengaging the fusion machinery [32]. In all, we conclude that acetate, but not hypertonic stress, likely affects PI signaling to stimulate vacuole fission.

Hypertonic stress induced by sorbitol was shown to block homotypic vacuole fusion in vitro by inactivating the Rab-GTPase Ypt7 [32]. Rab–GTPase inactivation was reported to promote fission by disrupting the interaction between the I-BAR protein Ivy1 and Ypt7 [31]. But it remains unclear if effects of hypertonic stress on vacuole fission are mediated by Ypt7 inactivation. To assess this possibility, we added rGyp1-46 –a recombinant, purified fragment of the Rab-GTPase Activating Protein (Rab-GAP) Gyp1 that inactivates Ypt7 by promoting GTP hydrolysis [32]–to vacuole fission reactions containing either KAc, KCl or KCl with 0.8 M glucose (Fig 4B). We found that increasing concentrations of rGyp1-46 had no effect on fission triggered by hypertonic stress, suggesting that Ypt7 was already inactivated under these conditions. In contrast, rGyp1-46 stimulated fission in presence of KAc, but had no effect on fission under isotonic conditions with KCl. These results suggest that (1) unlike hypertonic stress, acetate does not likely inactivate Ypt7 to promote fission, and (2) inactivating Ypt7 alone is incapable of triggering fission. The latter shows that fission and fusion can be independently regulated, suggesting that observed increases in fission likely reflect direct stimulation rather than the outcome of this process being unopposed when fusion is blocked. Notably, in vitro vacuole fission in response to KAc is blocked by recombinant Gdi1 protein, a Rab-GTPase chaperone that extracts Ypt7 from membranes [10]. Although this seems like a conflicting result, as extraction terminates Rab signaling, both observations support the existing idea that inactive Ypt7 must be present on membranes to engage and coordinate the machinery that mediates both fusion and fission, e.g. Ivy1 [31].

### Summary and conclusions

In sum, we justified use of a new sedimentation-based assay to study vacuole fission in vitro and find that acetate stress likely targets PI signaling whereas hypertonic stress likely inactivates Ypt7 to trigger fission while blocking homotypic fusion. However, inhibiting vacuolar PI-kinase(s) or Rab activity alone was insufficient to trigger vacuole fission in vitro, suggesting that engagement of multiple mechanisms by stressors is likely required to coordinate effects on organelle fission and fusion. Both stressors triggered fission of isolated vacuoles in the absence of cytosol and other cellular compartments, suggesting that all molecular machinery needed to sense and respond to acetate or osmotic stress is present on vacuole membranes. However, the identity of these mechanisms remains unresolved. Because acetate is a key intermediate of cellular metabolism [11], it is difficult to speculate but others have proposed that elevated levels may ultimately activate TOR signaling, which in turn supports vacuole fragmentation by possibly affecting PI signaling [10, 22]. As acetic acid, acetate may also impact compartmental pH and the activity of the V-type H^+^-ATPase that has been implicated in vacuole fission and fusion [25]. As for hypertonic stress, we speculate that it simply drives water efflux from the vacuole lumen leading to collapse of the membrane which would support scission. However, the precise mechanisms that mediate osmosis (e.g. a vacuolar aquaporin) and direct inactivation of Ypt7, to presumably engage the scission machinery, are unclear. Because this new fission assay can be easily scaled up to accommodate high-content screening experiments, it sets the stage for future studies that aim to test these hypotheses and further reveal the mechanisms underlying organelle fission and morphology in molecular detail.

## Materials and methods

### Yeast strains and reagents

We used the *S*. *cerevisiae* strain SEY6210 *pep4*Δ Vph1-GFP [*MATα leu2-3 ura3-52 his3-Δ200 trp1-Δ901 suc2-Δ9 lys2-801 pep4*::*HIS3 VPH1-GFP (TRP1)*] for the fluorescence-based in vitro fission assay and vacuole membrane detection by fluorescence microscopy [34]. BY4742 *pep4*Δ (*MATα leu2-3 ura3-52 his3-Δ200 lys2-801 pep4*::*NEO)* or *pho8*Δ (*MATα leu2-3 ura3-52 his3-Δ200 lys2-801 pho8*::*NEO) S*. *cerevisiae* strains purchased from Invitrogen (Carlsbad, CA, USA) were used for the in vitro fusion assay. All yeast growth media was purchased from BioShop Inc. (Burlington, ON, Canada). Buffer ingredients and reagents were purchased from Sigma Aldrich (St. Louis, MI, USA) with the exception of ficoll from GE Healthcare (Tokyo, Japan) and ATP from Roche (Indianapolis, IN, US). Recombinant Gyp1-46 protein and oxalyticase were expressed in *E*.*coli* and purified by affinity chromatography as previously described [32]. All proteins or reagents added to in vitro fusion or fission reactions were diluted in or buffer exchanged into PS buffer (20 mM PIPES, 200 mM sorbitol), aliquoted, flash frozen in liquid nitrogen and stored at –80 °C until use.

### Yeast vacuole isolation

Yeast cultures were grown in a shaking incubator overnight at 30 °C in 1 L YPD medium to a density of 1.4–1.8 OD600_nm_/mL. Cells were then harvested by centrifugation (3,000 g for 10 minutes at 4 °C), washed (10 minutes at 30 °C) with 50 mL buffer containing 100 μM DTT and 50 mM Tris-HCl pH 9.4, sedimented (3, 500 g for 5 minutes at room temperature), resuspended in 15 mL spheroplasting buffer (25 mM potassium phosphate pH 6.8 and 200 mM sorbitol in 1:20 YPD medium diluted in water) containing 1–2 μg/mL purified oxalyticase, and incubated for 30 minutes at 30 °C. Spheroplasts were collected by centrifugation (1,250 g for 2 minutes at 4 °C), resuspended in 2 mL ice-cold PS buffer (20 mM PIPES, 200 mM sorbitol) containing 15% ficoll, and treated with 0.2–0.4 μg/mL DEAE dextran for 3 minutes at 30 °C to disrupt the plasma membrane. Permeabilized spheroplasts were then transferred to an ultracentrifuge tube on ice, 8%, 4% and 0% ficoll layers were added on top, and samples were subjected to high-speed centrifugation (125,000 g for 90 minutes at 4 °C) to isolate vacuoles from other cell components. Vacuoles were then collected from interface between 4 and 0% ficoll layers and placed on ice until use. Vacuole protein concentrations were determined by Bradford assay.

### In vitro vacuole fission assay

To quantify vacuole membrane fission in vitro, we prepared 30 μL fission reactions by adding 6 μg of vacuoles isolated from SEY6210 *pep4*Δ Vph1-GFP cells to standard fission reaction buffer (PS buffer containing 5 mM MgCl_2_, 125 mM KCl, 10 mM CoA, and 1 mM ATP to stimulate fission; see [10]) and then incubated them at 27 °C for 30 minutes. Where indicated, increasing concentrations of glucose, sorbitol, wortmannin or recombinant Gyp1-46 protein were added, or KAc was replaced with KCl, prior to incubation. Reactions were then subjected to centrifugation (3,000 g for 3 minutes at 4 °C) to separate small vacuoles (present in the supernatant) from larger vacuoles (present in the pellet). After collecting the supernatant, pellets were resuspended in 20 μL fission reaction buffer and both samples were then transferred to a black conical-bottom 96-well microplate. GFP fluorescence (λ_ex_ = 485 nm, λ_em_ = 520 nm) was then measured using a Synergy H1 multimode microplate reader (BioTek Instruments Inc., Winooski, VT, USA), values were background subtracted and the ration of supernatant over total fluorescence was calculated as a measure of vacuole membrane fission in vitro. Data shown was normalized to the value obtained under control (no treatment), isotonic conditions. Reaction buffer osmolarity was confirmed using a Vapro 5520 vapor-pressure osmometer (Wescor, Logan, UT, USA). Vacuole diameter was measured using a Brookhaven 90 Plus Particle Size Analyzer (Brookhaven Instruments Cooperation).

### In vitro homotypic vacuole fusion assay

Homotypic vacuole fusion in vitro was measured using a colorimetric assay that relies on maturation of the alkaline phosphatase Pho8 [32]. In brief, 30 μL fusion reactions were prepared by adding 3 μg of vacuoles isolated from BY4742 *pho8*Δ cells and 3 μg of vacuoles isolated form BY4742 *pep4*Δ cells to standard fusion reaction buffer (PS buffer containing 125 mM KCl, 5 mM MgCl_2_, 10 μM CoA and 1 mM ATP to simulate fusion) and then incubated at 27 °C for 90 minutes. Where indicated increasing concentrations of sorbitol were added or KAc was replaced with KCl, prior to incubation. Upon membrane fusion, lumenal content mixing permits immature Pho8 (within vacuoles from cells missing Pep4) to be cleaved by the protease Pep4 (within vacuoles from cells missing Pho8) to activate the enzyme. Pho8 activity is then measured by adding 500 μL development buffer (250 mM Tris-HCl pH 8.5, 10 mM MgCl_2_, 0.4% triton X-100) containing 1 mM paranitrophenolphosphate, a Pho8 substrate, and incubated for 5 minutes at 30 °C. The phosphatase reaction was terminated with 500 μL stop buffer (100 mM glycine pH 11) and the absorbance of the yellow product, paranitrophenol, was measured at 400 nm using a NanoDrop 2000c spectrophotometer (Thermo Fisher Scientific, Waltham, MA, USA). A_400nm_ values were background subtracted and normalized to values obtained under control, isotonic conditions (125 mM KCl).

### Fluorescence microscopy

Using HILO (Highly Inclined and Laminated Optical sheet) microscopy, images of fission reactions containing vacuoles isolated from SEY6210 *pep4*Δ Vph1-GFP cells were acquired using a Nikon Eclipse TiE inverted microscope outfitted with a TIRF (Total Internal Reflection Fluorescence) illumination unit, Photometrics Evolve 512 EM-CCD camera, CFI ApoTIRF 1.49 NA 100x objective lens, and 50 mW 488 nm solid-state laser operated with Nikon Elements software (Nikon Canada Inc., Mississauga, ON, Canada). Images were acquired 1 μm into the sample. Micrographs shown were adjusted for brightness and contrast, inverted and sharpened with an unsharpen masking filter using Image J (National Institutes of Health, Bethesda, MD, USA) and Photoshop CC software (Adobe Systems, San Jose, CA, USA).

### Data analysis and presentation

All quantitative data was processed using Microsoft Excel software (Microsoft Corp., Redmond, WA, USA). Data was plotted using Kaleida Graph v.4.0 software (Synergy Software, Reading, PA, USA) and figure panels were prepared using Illustrator CC software (Adobe Systems, San Jose, CA, USA). Means ± S.E.M. are shown and Student’s two-tailed t-tests were used to assess significance (P < 0.05). Micrographs shown are best representatives of 5 biological replicates (each replicate represents a sample prepared from a separate yeast culture on different days), imaged at least 5 times each (technical replicates) whereby each field examined contained > 83 vacuoles. Fission and fusion data shown represent 3 or more biological replicates (each replicate represents a sample prepared from a separate yeast culture on different days) conducted in duplicate (technical replicates).
